# Kinetic contribution to extracellular Na^+^/K^+^ selectivity in the Na^+^/K^+^ pump

**DOI:** 10.1002/2211-5463.12418

**Published:** 2018-04-16

**Authors:** Elizabeth Vleeskens, Ronald J. Clarke

**Affiliations:** ^1^ School of Chemistry University of Sydney Australia; ^2^ The University of Sydney Nano Institute Australia

**Keywords:** ion selectivity, kinetic modelling, Na^+^,K^+^‐ATPase

## Abstract

The sodium potassium pump (Na^+^,K^+^‐ATPase) shows a high selectivity for K^+^ over Na^+^ binding from the extracellular medium. To understand the K^+^ selectivity in the presence of a high concentration of competing Na^+^ ions requires consideration of more than just ion binding affinities. Here, equilibrium‐based calculations of the extracellular occupation of the Na^+^,K^+^‐ATPase transport sites by Na^+^ and K^+^ are compared to fluxes through Na^+^ and K^+^ transport pathways. The results show that, under physiological conditions, there is a 332‐fold selectivity for pumping of K^+^ from the extracellular medium into the cytoplasm relative to Na^+^, whereas equilibrium calculations alone predict only a 7.5‐fold selectivity for K^+^. Thus, kinetic effects make a major contribution to the determination of extracellular K^+^ selectivity.

Selectivity for Na^+^ and K^+^ ions is crucial to animal physiology 1. Selective Na^+^ and K^+^ channels enable ion diffusion between the inside and outside of cells and are responsible for action potentials allowing nerve signal transmission and muscle contraction 1, 2. In Na^+^ channels, Na^+^ selectively binds from the extracellular medium where the concentration of Na^+^ is already high and the concentration of competing K^+^ ions is low. Similarly, K^+^ binds to K^+^ channels from the cytoplasm where the K^+^ concentration is already high and the concentration of competing Na^+^ ions is low.

In contrast, in the mechanism of the Na^+^,K^+^‐ATPase, K^+^ ions must bind from the extracellular medium, where the concentration of competing Na^+^ ions is much higher than that of K^+^. Therefore, the ion selectivity of the Na^+^,K^+^‐ATPase needs to be much more stringent than that of ion channels. In principle, there are two contributions to ion‐transporting selectivity: firstly, the equilibrium binding affinities of the enzyme for either Na^+^ or K^+^, and secondly, the kinetics of Na^+^‐ and K^+^‐ transporting pathways.

Information on the affinity of the Na^+^,K^+^‐ATPase for Na^+^ and K^+^ can be gained from the structure of the enzyme and theoretical free energy calculations. Recent crystal structures showed the Na^+^,K^+^‐ATPase in a transition state preceding the E1P.2Na^+^ conformation and a sodium occluded (Na_3_). E1P‐ADP state 3, 4. It was suggested that ionic radii play a role in selective binding of Na^+^ to the E1 state, especially to the third ion binding site, site III 4. Based on recent theoretical calculations, it has been suggested that ion hydration is a further important factor which determines the selectivity of Na^+^ channels for Na^+^ over K^+^ ions within the channel's selectivity filter 5. Previous crystal structures of the Na^+^,K^+^‐ATPase have shown the E2 conformation of the enzyme preferentially binds two Rb^+^ ions (as a K^+^ analogue) or two K^+^ ions 6, 7, 8. Based on a combination of structure‐based molecular dynamics simulations and electrophysiological experiments, Yu *et al*. 9. proposed that the protonation of key acidic amino acid residues of the Na^+^,K^+^‐ATPase is essential for K^+^ selectivity on the extracellular face of the protein. Structure‐based energy simulations can in principle explain differences in equilibrium ion binding affinities. However, as Yu *et al*. 9. pointed out, conductive selectivity of an ion pump could also be a result of kinetic factors. Furthermore, it is important to bear in mind that the Na^+^,K^+^‐ATPase is not in equilibrium and its ion pumping activity occurs on the millisecond–second timescale, a timeframe not currently accessible to molecular dynamics simulations, and that equilibrium‐based calculations of binding affinities to particular enzyme conformations are based on assumptions of slow subsequent reactions, that is, slow occlusion or dephosphorylation. Therefore, to fully understand the basis for the selective inward pumping of K^+^ by the Na^+^,K^+^‐ATPase, it is necessary to consider both the thermodynamics and the kinetics of the enzyme.

In the conventional Albers–Post scheme of Na^+^,K^+^‐ATPase activity, the E2P conformation of the enzyme releases 3Na^+^ ions to the extracellular medium per ATP hydrolysed and then takes up 2K^+^ ions, pumping them into the cell 10. Recently, a detailed kinetic model of ion pumping by the Na^+^,K^+^‐ATPase under physiological conditions based on the Albers–Post model was published 11 which included the exchange of 3Na^+^ ions for 2Na^+^ ions, that is, net one Na^+^ ion leaving the cell, in addition to the more well‐known 3Na^+^/2K^+^ exchange. Based on this model, kinetic calculations can be performed to predict the relative frequencies of 3Na^+^/2K^+^ versus 3Na^+^/2Na^+^ pumping, that is, to determine the overall extracellular ion selectivity of the Na^+^,K^+^‐ATPase. Here, the aim is to dissect the relative contributions of pure ion binding affinities (i.e., equilibrium effects) and kinetically induced perturbations of ion binding affinities due to subsequent reactions to the overall extracellular ion selectivity of the protein.

## Materials and methods

**Figure 1:**
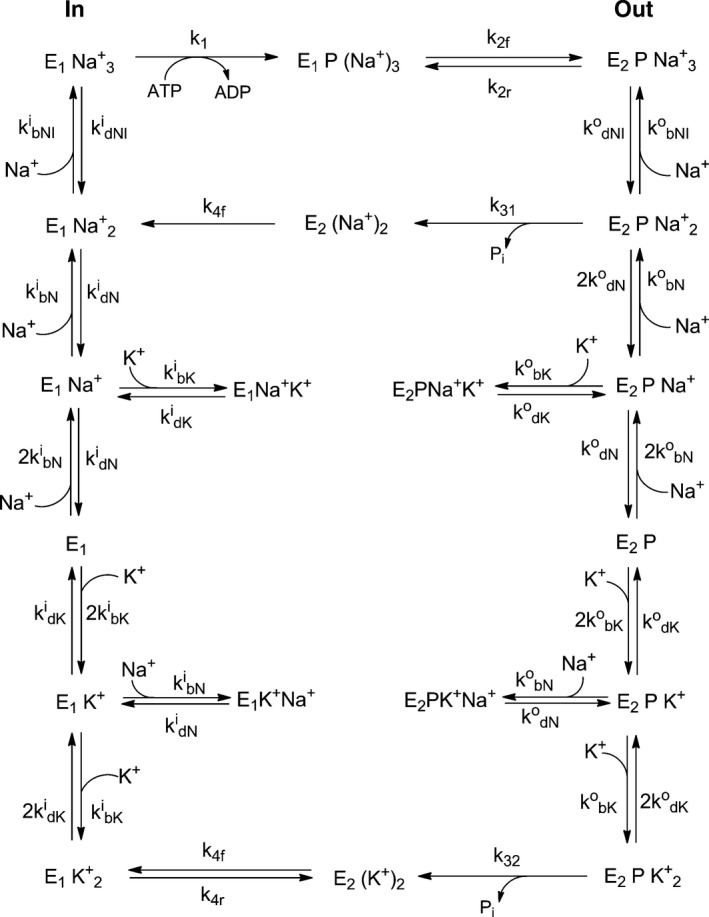
Albers–Post scheme describing the Na^+^,K^+^‐ATPase catalytic mechanism. Reactions (1) and (2) (i.e., ATP phosphorylation and the E1P‐E2P conformational change of phosphorylated protein) are coupled to reactions (3) and (4) (i.e., dephosphorylation and the E2‐E1 conformational change of dephosphorylated protein) by a series of vertically drawn ion binding and dissociation reactions on the cytoplasmic face of the protein (superscript i, on the left) and on the extracellular face (superscript o, on the right). Two possible enzymatic pathways are considered, with 3Na^+^ ions being pumped from the cytoplasm to the extracellular medium in exchange for either 2Na^+^ ions (Na^+^/Na^+^ exchange mode) or 2K^+^ ions (Na^+^/K^+^ exchange mode).

Computer simulations of the steady‐state Na^+^,K^+^‐ATPase turnover were performed using the commercially available program berkeley‐madonna 8.0 (University of California at Berkeley, Berkeley, CA, USA) and the variable step‐size Rosenbrock integration method for stiff systems of differential equations. The simulations yielded the time course of the concentration of each enzyme intermediate involved and the steady‐state flux through each of the enzyme's parallel pathways. For the purposes of simulations, each enzyme intermediate was normalized to a unitary concentration and the enzyme was assumed arbitrarily to be initially totally in the E1 state. To determine the steady‐state flux through each pathway, each simulation was carried out until the distribution between the different enzyme states no longer changed and the fluxes reached constant values. The entire kinetic model is shown in Fig. 1.

## Results and Discussion

Kinetic simulations based on the model shown in Fig. 1, which has been described in detail previously 11, predict that under physiological conditions, 3Na^+^/2K^+^ pumping accounts for 99.7% of all ion exchanges carried out by the Na^+^,K^+^‐ATPase, whereas 3Na^+^/2Na^+^ pumping only accounts for 0.3%. The kinetic simulations do not take into account dephosphorylation by the back transfer of phosphate to ADP, which has only been demonstrated 12 to occur appreciably under nonphysiological conditions of high external Na^+^ and no external K^+^. Taking the ratio of the two percentages given above amounts to a K^+^ inward transport preference over Na^+^ of 332 : 1. In the absence of any preference for either ion, that is, simply based on the extracellular ion concentrations 13, the ratio would be 0.0286 : 1 (4 mm K^+^; 140 mm Na^+^). Thus, equilibrium and kinetic factors together in fact produce an enhancement of the relative frequencies of K^+^ inward transport over Na^+^ by a factor of 11 620. The kinetic simulations show that the 3Na^+^/2K^+^ pathway is almost exclusively favoured under physiological conditions. The question is whether binding affinities alone can account for the high preference for the 3Na^+^/2K^+^ pathway or whether kinetic factors play an important role.

To determine the contribution of ion binding affinities to extracellular ion selectivity of the Na^+^,K^+^‐ATPase, the degrees of occupancy of all of the E2P forms of the enzyme need to be determined, because this state of the enzyme has the ion binding sites facing the extracellular medium (Fig. 1). Na^+^ and K^+^ bindings to the E2P state are described by the following series of seven equilibria:

$${\text{E2PNa}}_{3}^{+}\overset{K_{N1}^{o}}{⇄}{\text{E2PNa}}_{2}^{+}+{\text{Na}}^{+}$$

$${\text{E2PNa}}_{2}^{+}\overset{K_{N}^{o}}{⇄}{\text{E2PNa}}^{+}+{\text{Na}}^{+}$$

$${\text{E2PNa}}^{+}\overset{K_{N}^{o}}{⇄}\text{E2P}+{\text{Na}}^{+}$$

$${\text{E2PK}}^{+}\overset{K_{K}^{o}}{⇄}\text{E2P}+K^{+}$$

$${\text{E2PK}}_{2}^{+}\overset{K_{K}^{o}}{⇄}{\text{E2PK}}^{+}+K^{+}$$

$${\text{E2PNa}}^{+}K^{+}\overset{K_{K}^{o}}{⇄}{\text{E2PNa}}^{+}+K^{+}$$

$${\text{E2PNa}}^{+}K^{+}\overset{K_{N}^{o}}{⇄}{\text{E2PK}}^{+}+{\text{Na}}^{+}$$

The terms $K_{N1}^{o}$, $K_{N}^{o}$, etc., represent here the equilibrium dissociation constants for each equilibrium.

The fraction of the total enzyme in the ${\text{E2PK}}_{2}^{+}$, E2PK^+^, ${\text{E2PNa}}_{3}^{+}$, ${\text{E2PNa}}_{2}^{+}$, E2PNa^+^ and E2P states relative to all possible E2P states are given by Eqns (1), (2), (3), (4), (5), (6), (7), respectively, where the denominator, *D*, in each equation is given by Eqn (8).

(1) $$f\left({\text{E2PK}}_{2}^{+}\right)=\left({\left[K^{+}\right]}_{o}^{2}/K_{K}^{o2}\right)/D$$

(2) $$f\left({\text{E2PK}}^{+}\right)=\left(2{\left[K^{+}\right]}_{o}/K_{K}^{o}\right)/D$$

(3) $$f\left({\text{E2PNa}}_{3}^{+}\right)=\left({\left[{\text{Na}}^{+}\right]}_{o}^{3}/K_{N1}^{o}K_{N}^{o2}\right)/D$$

(4) $$f\left({\text{E2PNa}}_{2}^{+}\right)=\left({\left[{\text{Na}}^{+}\right]}_{o}^{2}/K_{N}^{o2}\right)/D$$

(5) $$f\left({\text{E2PNa}}^{+}\right)=\left(2{\left[{\text{Na}}^{+}\right]}_{o}/K_{N}^{o}\right)/D$$

(6) f(E2P)=1/D

(7) $$f\left({\text{E2PK}}^{+}{\text{Na}}^{+}\right)=\left(4{\left[K^{+}\right]}_{o}{\left[{\text{Na}}^{+}\right]}_{o}/K_{K}^{o}K_{N}^{o}\right)/D$$

(8) $$\begin{array}{cc} & D=1+2{\left[K^{+}\right]}_{o}/K_{K}^{o}+{\left[K^{+}\right]}_{o}^{2}/K_{K}^{o2}+2{\left[{\text{Na}}^{+}\right]}_{o}/K_{N}^{o}\\ & +{\left[{\text{Na}}^{+}\right]}_{o}^{2}/K_{N}^{o2}+{\left[{\text{Na}}^{+}\right]}_{o}^{3}/K_{N1}^{o}K_{N}^{o2}+4{\left[K^{+}\right]}_{o}{\left[{\text{Na}}^{+}\right]}_{o}/K_{K}^{o}K_{N}^{o} \end{array}$$

Based on Eqns (1), (2), (3), (4), (5), (6), (7), (8), the fraction of enzyme in each E2P state (Table 1) was calculated using experimentally determined dissociation constants, some of which are voltage dependent^14^, ^15^, ^16^, ^17^ and typical extracellular ion concentrations of [Na^+^]_o_ = 140 mm and [K^+^]_o_ = 4 mm 13. The voltage across the cell membrane, *V*_m_, at 24 °C was taken to be −0.079 V, previously calculated from the Goldman–Hodgkin–Katz (GHK) equation 11. The values of the dissociation constants used in the calculations were determined using Na^+^,K^+^‐ATPase derived from kidney tissue, which occurs as the α_1_β_1_ isoform of the enzyme. The values of the dissociation constants used were $K_{N1}^{o}$ = 13.4 mm, $K_{N}^{o}$ = 128 mm and $K_{K}^{o}$ = 0.424 mm. These values were calculated from the corresponding values at *V*_m_ = 0 mV^13^ using the Boltzmann expression K* *= K_*V*=0_exp(*aFV*_m_/*RT*). The dielectric coefficients, *a*, for $K_{N1}^{o}$, $K_{N}^{o}$ and $K_{K}^{o}$ were taken to be 0.65, 0.37 and 0.37, respectively 14.

**Table 1:** Percentages of the total Na^+^,K^+^‐ATPase in each of the possible E2P states at 24 °C based on equilibrium calculations

Enzyme state	Percentage of total enzyme
${\text{E2PK}}_{2}^{+}$	20.04
E2PK^+^	13.33
${\text{E2PNa}}_{3}^{+}$	27.68
${\text{E2PNa}}_{2}^{+}$	2.66
E2PNa^+^	4.86
E2P	2.21
E2PK^+^Na^+^	29.21

From the results shown in Table 1, it can be seen that the percentage of the enzyme in the ${\text{E2PK}}_{2}^{+}$ conformation, that is, the state immediately prior to occlusion of 2K^+^ and dephosphorylation of the enzyme (Fig. 1), relative to all possible E2P states, is 20.04%. The 20.04% K^+^ transport accounted for by selective binding can be compared with the results obtained with the kinetic model, which predicted that under physiological conditions, 99.7% of the pumping cycles should be of the 3Na^+^/2K^+^ type, with only 0.3% due to 3Na^+^/2Na^+^ exchange. Analysing the data in a slightly different way, equilibrium binding predicts that 2.66% (Table 1) of the total E2P pool of enzyme would be present in the ${\text{E2PNa}}_{2}^{+}$ state, capable of transporting Na^+^ back across the membrane, whereas 20.04% would be present as ${\text{E2PK}}_{2}^{+}$, that is, a 7.5‐fold greater occupancy by 2K^+^ than 2Na^+^. In comparison, the kinetic simulations (see earlier) predict a 332‐fold preference for K^+^ transport over Na^+^.

Thus, kinetic factors are crucial to enhancing extracellular K^+^ selectivity to the level of virtual exclusive inward K^+^ pumping. These kinetic factors include the following: (a) occlusion of Na^+^ or K^+^ from the ${\text{E2PNa}}_{2}^{+}$ or ${\text{E2PK}}_{2}^{+}$ states, respectively, (b) much more rapid extracellular K^+^ occlusion than Na^+^, and (c) subsequent dephosphorylation, which continually perturb the extracellular ion binding equilibria and effectively siphon virtually all of the enzyme through the 3Na^+^/2K^+^ pathway.

The effect of the extracellular Na^+^ and K^+^ occlusion pathways on the perturbation of the E2P binding equilibria can be assessed by simulating the enzyme turnover using the kinetic model 11, but with the occlusion rate constants for each ion set to equal values. This alone predicts that 80.7% of the cycles would involve 3Na^+^/2K^+^ exchange and only 19.3% 3Na^+^/2Na^+^ exchange. The preference for 3Na^+^/2K^+^ exchange can be explained in part due to the fact that only the ${\text{E2PNa}}_{2}^{+}$ and ${\text{E2PK}}_{2}^{+}$ states are considered to undergo ion occlusion. Both of these reactions cause a shift away from the ${\text{E2PNa}}_{3}^{+}$ state, hence facilitating K^+^ binding. Assuming the mixed state E2PK^+^Na^+^ is incapable of ion occlusion, its occupation would also be depleted by both the Na^+^ and K^+^ occlusion pathways. The further increase in the preference for 3Na^+^/2K^+^ exchange from 80.7% to 99.7% is achieved by a much more rapid ion occlusion by ${\text{E2PK}}_{2}^{+}$ than ${\text{E2PNa}}_{2}^{+}$. Thus, the ion binding equilibria are shifted even further in the direction of ${\text{E2PK}}_{2}^{+}$.

In summary, although the E2P conformation of the Na^+^,K^+^‐ATPase does bind K^+^ ions extracellularly much more strongly than Na^+^ ions, because of the high degree of competition from the much higher concentration of Na^+^ ions in the extracellular medium relative to K^+^, the stronger K^+^ binding affinity alone is insufficient to explain the virtual exclusive pumping of K^+^ ions from the extracellular medium into the cytoplasm under physiological conditions. As recently pointed out by Rui *et al*. 18, the Na^+^,K^+^‐ATPase overcomes this problem by having a much higher activation energy barrier for the occlusion of Na^+^ than K^+^. Rui *et al*. 18. referred to this as a ‘self‐correcting occlusion’ mechanism. Thus, kinetics also makes a major contribution towards the enzyme's extracellular selectivity for K^+^. After K^+^ ions bind to the E2P conformation, a conformational change of the protein occurs, occluding the ions within the protein matrix so that they have no access to either the extracellular medium or the cytoplasm. Although this conformational change can also occur when Na^+^ binds, presumably because of the larger size of the K^+^ ion and its ability to coordinate with surrounding amino acid side chains, the reaction is much faster with K^+^ ions present in the binding sites. The rate constant for K^+^ occlusion by the E2P conformation has been experimentally determined to be 342 s^−1^ 19, whereas Na^+^ occlusion occurs with a rate constant of only around 4 s^−1^ 16, 20. Under physiological conditions, the faster occlusion of K^+^ ions draws virtually all of the enzyme cycles through the K^+^ pumping pathway in spite of an expected equilibrium occupation of the E2P conformation by K^+^ of only 20%.

It is worth mentioning that the finding here that kinetics plays a major role in determining the extracellular selectivity of the Na^+^,K^+^‐ATPase for K^+^ is also consistent with experimental measurements in which the enzyme was treated with palytoxin (PTX) 21. PTX is a marine toxin which binds to the Na^+^,K^+^‐ATPase and turns it from a pump into a channel by stabilizing an open ion transport pathway across the entire membrane 22, 23. Because the Na^+^,K^+^‐ATPase with PTX bound still hydrolyses ATP, Harmel and Apell 21 were able to measure the *K*_m_ of the protein for external Na^+^ and K^+^ in the PTX bound and free states. If one uses 1/*K*_m_ as an approximation of binding affinity, their results indicated that treatment with PTX causes a 22‐fold reduction in the selectivity of the E2P state for K^+^ over Na^+^. This could be explained by the inability of the PTX‐treated enzyme to preferentially occlude K^+^ ions, thus in qualitative agreement with the results reported here, stressing the importance of occlusion in determining ion selectivity.

## Author contributions

RJC designed the project, developed the theory and wrote the paper. EV performed simulations.

## References

[1] Hille B (2001) Ionic Channels of Excitable Membranes, 3rd edn Sinauer Associates Inc., Sunderland, MA.

[2] Doyle DA , Morais Cabral J , Pfuetzner RA , Kuo A , Gulbis JM , Cohen SL , Chait BT and MacKinnon R (1998) The structure of the potassium channel: molecular basis of K^+^ conduction and selectivity. Science 280, 69–77. 9525859 10.1126/science.280.5360.69

[3] Nyblom M , Poulsen H , Gourdon P , Reinhard L , Andersson M , Lindahl E , Fedosova N and Nissen P (2013) Crystal structure of the Na^+^, K^+^‐ATPase in the Na^+^‐bound state. Science 342, 123–127. 24051246 10.1126/science.1243352

[4] Kanai R , Ogawa H , Vilsen B , Cornelius F and Toyoshima C (2013) Crystal structure of a Na^+^‐bound Na^+^, K^+^‐ATPase preceding the E1P state. Nature 502, 201–206. 24089211 10.1038/nature12578

[5] Corry B and Thomas M (2012) Mechanism of ion permeation and selectivity in a voltage‐gated sodium channel. J Am Chem Soc 134, 1840–1846. 22191670 10.1021/ja210020h

[6] Morth JP , Pedersen BP , Toustrup‐Jensen MS , Sørensen TL‐M , Petersen J , Andersen JP , Vilsen B and Nissen P (2007) Crystal structure of the sodium potassium pump. Nature 450, 1043–1050. 18075585 10.1038/nature06419

[7] Laursen M , Yatime L , Nissen P and Fedosova NU (2013) Crystal structure of the high‐affinity Na^+^, K^+^‐ATPase‐ouabain complex with Mg^2+^ bound in the cation binding site. Proc Natl Acad Sci USA 110, 10958–10963. 23776223 10.1073/pnas.1222308110 PMC3704003

[8] Shinoda T , Ogawa H , Cornelius F and Toyoshima C (2009) Crystal structure of the sodium‐potassium pump at 2.4 Å resolution. Nature 459, 446–450. 19458722 10.1038/nature07939

[9] Yu HB , Ratheal I , Artigas P and Roux B (2012) Molecular mechanisms of K^+^ selectivity in Na/K pump. Aust J Chem 65, 448–456.

[10] Post RL , Hegyvary C and Kume S (1972) Activation by adenosine triphosphate in the phosphorylation kinetics of sodium and potassium ion transport adenosine triphosphate. J Biol Chem 247, 6530–6540. 4263199

[11] Clarke RJ , Catauro M , Rasmussen HH and Apell H‐J (2013) Quantitative calculation of the role of the Na^+^, K^+^‐ATPase in thermogenesis. Biochim Biophys Acta 1827, 1205–1212. 23850548 10.1016/j.bbabio.2013.06.010

[12] Garrahan PJ and Glynn IM (1966) Driving the sodium pump backwards to form adenosine triphosphate. Nature 211, 1414–1415. 5969842 10.1038/2111414a0

[13] Kong BY and Clarke RJ (2004) Identification of potential regulatory sites of the Na^+^, K^+^‐ATPase by kinetic analysis. Biochemistry 43, 2241–2250. 14979720 10.1021/bi0355443

[14] Garcia A , Rasmussen HH , Apell H‐J and Clarke RJ (2012) Kinetic comparisons of heart and kidney Na^+^,K^+^‐ATPases. Biophys J 103, 677–689; and correction *Biophys J* (2013) **104**, 1214. 22947929 10.1016/j.bpj.2012.07.032 PMC3443779

[15] Wuddel I and Apell H‐J (1995) Electrogenicity of the sodium transport pathway in the Na, K‐ATPase probed by charge pulse experiments. Biophys J 69, 909–921. 8519991 10.1016/S0006-3495(95)79965-9 PMC1236320

[16] Kane DJ , Grell E , Bamberg E and Clarke RJ (1998) Dephosphorylation kinetics of pig kidney Na^+^, K^+^‐ATPase. Biochemistry 37, 4581–4591. 9521778 10.1021/bi972813e

[17] Babes A and Fendler K (2000) Na^+^ transport, and the E_1_P‐E_2_P conformational transition of the Na^+^/K^+^‐ATPase. Biophys J 79, 2557–2571. 11053130 10.1016/S0006-3495(00)76496-4 PMC1301138

[18] Rui H , Artigas P and Roux B (2016) The selectivity of the Na^+^/K^+^‐pump is controlled by binding site protonation and self‐correcting occlusion. Elife 5, e16616. 27490484 10.7554/eLife.16616 PMC5026471

[19] Myers SL , Cornelius F , Apell H‐J and Clarke RJ (2011) Kinetics of K^+^ occlusion by the phosphoenzyme of the Na^+^, K^+^‐ATPase. Biophys J 100, 70–79. 21190658 10.1016/j.bpj.2010.11.038 PMC3010830

[20] Hobbs AS , Albers RW and Froehlich JP (1980) Potassium‐induced changes in phosphorylation and dephosphorylation of the (Na^+^ + K^+^)‐ATPase observed in the transient state. J Biol Chem 255, 3395–3402. 6245079

[21] Harmel N and Apell H‐J (2006) Palytoxin‐induced effects on partial reactions of the Na,K‐ATPase. J Gen Physiol 128, 103–118. 16801384 10.1085/jgp.200609505 PMC2151552

[22] Artigas P and Gadsby DC (2003) Ion occlusion/deocclusion partial reactions in individual palytoxin‐modified Na/K pumps. Ann N Y Acad Sci 986, 116–126. 12763784 10.1111/j.1749-6632.2003.tb07148.x

[23] Artigas P and Gadsby DC (2003) Na^+^/K^+^‐pump ligands modulate gating of palytoxin‐induced ion channels. Proc Natl Acad Sci USA 100, 501–505. 12518045 10.1073/pnas.0135849100 PMC141024

